# The potential of bicycle commuting to reduce carbon emissions in Finland

**DOI:** 10.1371/journal.pone.0335010

**Published:** 2025-11-13

**Authors:** Emilia Suomalainen, Marko Tainio

**Affiliations:** 1 Finnish Environment Institute, Helsinki, Finland; 2 Systems Research Institute, Polish Academy of Sciences, Warsaw, Poland; The University of Tokyo, JAPAN

## Abstract

There is an increasing amount of evidence that cycling is an effective way to decarbonise everyday mobility. The potential of cycling is however less well understood in cold climates, where seasonal weather conditions are seen as a major obstacle. This work explores the potential of cycling to substitute for car use on commute trips in Finland. A binary logistic regression model is first built based on national travel survey data to describe cycling behaviour on home–work trips according to trip distance, hilliness, temperature, snow cover, gender of the cyclist, car availability, and city region. This model is then used to quantify cycling uptake scenarios and estimate cycled mileage, replaced car travel, and climate emission reductions. E-bike scenarios are also explored. The results indicate that it would be possible to set ambitious targets for cycling uptake, even doubling the mileage cycled, leading to non-negligible emission reductions.

## Introduction

Finland has an objective to become a carbon-neutral welfare society by 2035 [1], with national actions for the road transport sector described in the Roadmap to fossil-free transport [2]. For active travel, i.e., cycling and walking, Finland is targeting a 30% increase in the number of trips by 2030 [3]. The current modal share of cycling and walking is approximately 23% and 7% respectively [4]. This target is notably supported by national subventions to cities and municipalities for the development of cycling infrastructure. Other policies implemented included a ‘cash for clunkers’ scheme [5], which could be used to acquire an electric bicycle. Currently, a company bike benefit scheme is also boosting the demand for e-bikes. In recent years, e-bike sales have been rising rapidly in Finland, and this development has continued even after the Covid-19 pandemic [6].

**Fig 1:**
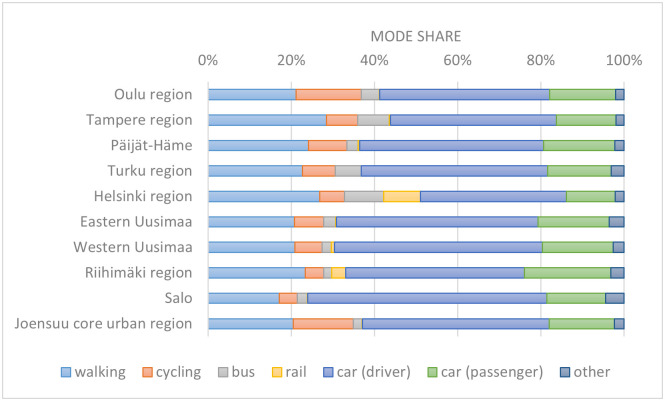
Modal shares in Finland’s city regions. Mode share on domestic trips under 100 km [8].

The national active travel targets are relatively modest and reflect the general opinion that it would be difficult to increase cycling significantly in Finland. It is thought that the harsh winter conditions, with icy and snowy roads, largely prevent cycling during wintertime. Furthermore, recent transport and land use trends, such as increasing commute distances from 2000 to 2015, decreasing potential for the use of sustainable transport modes, tendency of jobs to move outside walkable city centres, and increasing car dependency [7] have called into question the potential of active mobility to decrease climate emissions. There are however major differences between city regions: for example, Oulu and Joensuu boast higher levels of cycling than other cities in Finland (**Fig 1**). Oulu is especially famous for its high levels of winter cycling that are thought to be due to good winter maintenance. Both cities are also well-known for their excellent cycling infrastructure.

Finland is a small country in Northern Europe home to approximately 5.6 million inhabitants. There are four seasons, and the winter season with sub-zero temperatures lasts from three to seven months depending on the latitude [9]. While the transport system is heavily car-based—62% of all trips and 84% of mileage are by car as driver or passenger [4]—bicycle ownership is also quite common, estimated at over 80% [10]. Though 74% of the inhabitants live in city regions [11], and especially in the Southwestern corner of the country, the relationship to nature is important to many. There is only one metropolitan region, the Helsinki Metropolitan Area, that has a population of over a million inhabitants. At the national level, the population density is low, leaving a large area to green surfaces, notably forests. The population is aging rapidly, and population growth depends heavily on migration. While Finland generally has high ratings in international gender equality indices, there are gender differences both in mobility and in employment. For instance, women tend to travel shorter distances, and men drive more often while women walk or use public transport more [4]. In Finland, 60% of cycled trips are made by women [12]. The employment rate of women is slightly below that of men, and more women have part-time jobs [13]. Gendered segregation is also visible in work life as many sectors are either female or male-dominated [14].

In this context, it is interesting to take a closer look at the potential of cycling to decarbonise transport in Finland. How ambitious is the national +30% target for active travel when applied to cycling? As there are city regions in Finland where cycling is more common than elsewhere, what would happen if the conditions in these places could be generalized to other city regions in Finland? What would this mean in terms of number of trips and cycled mileage? Given that the carless population cycles more than car users, what would happen if more car drivers might be persuaded to adopt the same behaviour? In addition, the rising sales of pedal-assist e-bikes are changing cycling behaviour and offer new opportunities for decarbonising mobility. Are the potential emission reductions offered by e-bikes significant on a national scale? What is the potential of cycling to replace car travel, and would cycling merit greater attention when thinking of transport decarbonisation in Finland?

We set out to explore these questions with a focus on commute trips. Commuting is to a large extent car-based in Finland: nearly 70% of trips to work are made by car and only about 10% by bicycle, and cycling corresponds to a mere 3% of the commute mileage [15]. Commuting to work constitutes an important share of constrained travel and is therefore a great target for increasing cycling uptake. Work and education related trips make up 25% of all domestic trips and 28% of mileage in Finland [16]. 55% of all cycled trips are at most two kilometres long and 19% of cycled trips are commute trips [12]. The average length of trips cycled to work is 4.4 kilometres, though men tend to cycle longer distances than women [12]. For the working-age population (18–64-year-olds), trips to work constitute at least 20% of trips in all age groups [16]. Contrary to many other utilitarian trips, such as shopping, the destination (i.e., the workplace) is to a large extent immutable and thus the trip distance is fixed.

The aim of this study is to estimate the CO_2_ emission reduction potential of cycling to work in Finland. To do this, we build a logistic regression model and define ‘what if’ scenarios for cycling uptake based on current cycling behaviour. Our research questions are: what is the potential of cycling to reduce emissions from car use on trips to work in Finland’s city regions? In particular, a) what would be the impact (in terms of number of trips, mileage, substitution of travel by car, and climate emissions) if more city regions displayed the same cycling behaviour as those living in the top cycling region in Finland, Oulu; b) what would be the impact if everyone cycled like the carless population; and c) what kind of an impact might the large-scale adoption of e-bikes have? Lastly, what policy implications can be derived from the model results, notably in terms of national cycling targets and the emission reduction potential of cycling?

### Relevant literature

Cycling has many benefits from positive health impacts to reducing air pollution and greenhouse gas emissions, especially when compared to private cars. Although most people cycle relatively short distances, there is an increasing amount of evidence that cycling can contribute significantly to decarbonising transport. Based on data from seven European cities, Brand et al. [17] found that for daily travel activities cyclists’ life-cycle CO_2_ emissions were 84% lower than the emissions of those who did not cycle. For an average person, shifting travel modes from car to cycling on one trip per day for 200 days per year would decrease life-cycle CO_2_ emissions by about 0.5 tCO_2_ per year [18]. Active travel is not just additional travel; it can substitute for motorised travel and, even though all car trips cannot be substituted, the emission reduction potential of active travel is considerable [18]. There is also strong evidence that motorised transport imposes significant health costs on society and that more active travel would bring significant health and economic benefits (see, e.g., Gössling et al. [19]). Even when taking into account exposure to air pollution, the health benefits of cycling and walking outweigh the risks in most urban environments [20]. Better understanding cycling behaviour and increasing cycling levels is therefore interesting from both an environmental and a public health point of view.

In comparison to conventional bicycles, electric bicycles or e-bikes extend the range of destinations attainable and make cycling accessible to a larger share of the population. In this way, e-bikes can bring even more sizeable emission benefits. It has been estimated in England that by substituting travel by private car e-bikes might reduce life-cycle greenhouse gas emissions by up to 24 MtCO_2_ per year and tailpipe emissions by up to 14 MtCO_2_ per year [21]. The CO_2_ savings per person were the highest in rural settings, or over 750 kg CO_2_ per person per year [21]. Another study for England and Wales estimated a reduction of transport emissions by nearly 860 ktCO_2_eq per year for commute trips in an e-bike scenario [22]. In China, it was estimated that shared e-bikes might reduce carbon emissions from urban transport by 3.3% [23]. The emission impacts of e-bikes have also been estimated by analysing mode displacement based on survey data (see, e.g., Fyhri et al. [24] for Norway; Winslott Hiselius and Svensson [25] for Sweden). It has been shown that in addition to contributing to transport decarbonisation and a mode shift, e-bike riding is moderate to vigorous intensity physical activity [26]. Although e-biking reduces the use of conventional bicycles [27,28], e-bikes increase both the frequency and duration of cycling trips [29]. E-bikes have also been gaining attention in the context of decarbonising car-dependent rural areas [30].

Given these positive impacts of cycling both in terms of carbon emissions and health, it is of interest to estimate the potential for increasing cycling uptake. Modelling studies on cycling potential can be found for instance for England and Wales, where Lovelace et al. [31] and Woodcock et al. [22] developed a methodology called the Propensity to Cycle Tool [32] and estimated the health and carbon impacts of increased commute cycling. The health and emission impacts of a mode shift to cycling have also been estimated in New Zealand [33]. In a slightly different vein, Oviedo and Sabogal-Cardona [34] recently estimated the potential for a mode shift to cycling in Bogotá based on travel distance and time. In particular, the potential of e-bikes to replace travel by car has garnered much attention in recent years as electric bicycle sales continue to rise. McQueen et al. [35] used North American survey data to develop a mode displacement model and applied it to Portland to estimate the greenhouse gas emission impact of e-bikes. Several studies also analyse mode shift potential without quantifying the related emission benefits (see, e.g., Sun et al. [28] for the Netherlands). There are also studies that take a more heavily modelling-based approach. Philips et al. [21] estimated the emission reduction potential of e-bikes using National Travel Survey data for England: they built a synthetic population to model the physical capabilities of travelling by e-bike. Bucher et al. [36] estimated the potential of e-bikes to bring emission reductions on trips to work in Switzerland by using a commuter matrix and defining criteria for e-bike use in different weather conditions (maximal precipitation and minimal temperature). They also considered the impact of elevation on temperature and the maximal duration of the e-bike trip. In a similar vein, Gebhardt et al. [37] estimated the car trip substitution potential of e-scooters using German mobility data. They used weather-related criteria (no heavy rain, snowfall, or icy roads) to filter potential trips.

The impact of weather conditions on cycling has been explored in numerous studies. For instance, temperature, precipitation, and wind are generally found to impact cycling levels, although the effects vary from one region to another [38,39]. Darkness is another potential factor [39]. It has also been shown that utilitarian cycling is generally less sensitive to weather conditions than recreational cycling [38,40]. It has however been pointed out that research has often focused on temperate and hotter climates and that cycling in cold climate winter conditions remains less well understood [41]. Several works exploit regression to model the impact of weather on cycling. For instance, Thomas et al. [40] used regression modelling with weather variables to explain the fluctuation of cycling demand in the Netherlands. The weather elasticity of cycling has also been explored in Germany by Goldmann and Wessel [42] using a composite adverse weather indicator. Guidon et al. [43] employed negative binomial regression to model the impact of weather and the day of the week on travel demand in a free-floating e-bike sharing system in Zurich. In the Netherlands, de Kruijf et al. [44] showed using multilevel binary logistic regression that e-cycling to work was impacted by air temperature, rain, heavy winds, and snow/iciness.

While the German and Swiss studies on micro-mobility potential by Gebhardt et al. [37] and Bucher et al. [36] take the impact of weather into account, it is done in a binary manner: weather conditions are either favourable for micromobility or not. To our knowledge, more realistic modelling of the impact of seasons and weather conditions has seldom been included in an estimation of cycling potential, except in a previous work by the authors that focused on modelling cycling to school [45]. In Sweden, one study estimated the potential impact of increased winter maintenance based on a categorisation of commute cyclists according to cycling frequency and seasonality [46]. In Finland, cycling levels are greatly dependent on the season: in summer, the number of commute trips by bicycle per person and per day is over four times greater and cycled mileage six times greater than in winter [15]. The inclusion of seasonality in an assessment of cycling potential in cold climate countries is therefore crucial.

The Propensity to Cycle Tool appears as a suitable starting point for modelling cycling potential as it takes the main environmental factors impacting cycling, namely trip length and hilliness, into account in a continuous manner [31]. This tool exploits a binary logistic regression model that expresses the propensity that a given trip is cycled according to trip distance and its gradient or hilliness [31]. The methodology of the Propensity to Cycle Tool can be applied at individual or trip level [22], and it can be used to build scenarios describing increased cycling uptake and calculate their emission impacts by modelling how new cycling trips replace trips previously made by car. Seasonal changes in cycling behaviour are however not taken into account. In this work, the Propensity to Cycle methodology is therefore adapted to include the impact of the seasons in order to explore how commute cycling changes over the year and especially in winter conditions. This is particularly relevant in countries with cold climates.

In building our logistic regression model, other factors that impact cycling levels were also considered. Heinen et al. [47] provide an overview of factors influencing bicycle commuting. They categorized the determinants into five groups: built environment, natural environment (including climate and weather conditions), socio-economic factors, psychological factors, and other factors (i.e., cost, time, effort, and safety). Long trips, hilliness, and car ownership were found to be negatively associated with cycling while weather conditions, such as low temperatures, also have an impact [47]. Interactions between gender and weather conditions have also been observed, with lack of daylight and rain being greater barriers to women’s cycling [47]. In addition to built environment and cycling infrastructure, the importance of a positive attitude towards cycling has been underlined in some studies, especially for people with cars [48]. For commuter cyclists, the presence of bicycle storage facilities and changing rooms also has a positive impact [49]. Kroesen and Handy [50] found based on a Dutch mobility panel that, in addition to the distance to work, travel allowances can impact bicycle commuting. The impact of socio-demographic variables varies from country to country, and the effects of age and income levels are ambiguous [49]. Unfortunately, the Finnish National Travel Survey data [8] used for building our model lacks many of the variables described above. The data and the model building process are described in Materials and methods.

To summarise, the contributions of this work are three-fold. Firstly, this work is of strong practical and transport policy interest as the carbon emission savings of increased cycling uptake have not yet been the object of a scientific study in Finland. In addition, the potential for increasing commute cycling has also not yet been assessed in the country. Secondly, this work aims to provide a methodological contribution to the literature by adapting a previously developed methodology from the Propensity to Cycle Tool to a country with strong seasonality in cycling. Thirdly, our results highlight interesting interactions between cold climate winter conditions and other variables (trip distance and gender) that have been little explored in the literature.

## Materials and methods

### Logistic regression model

To model cycling behaviour on commute trips in Finland, our starting point was the binary logistic regression model described by Lovelace et al. [31]. This model explains the propensity to cycle—the proportion of commuters that cycle to work on a given origin–destination pair—based on trip distance and hilliness. Both linear and non-linear (square and square root) terms for the trip distance were included in the original model, as cycling probability first increases with distance, peaking at around 2–3 kilometres, and then decreases with a long tail. The UK model is used to describe cycling behaviour at the individual level based on the most often used means of commuting, and it does not take the impact of weather conditions into account. As cycling levels show significant seasonal variations in Finland [15,51], the UK model was adapted to the Finnish context by, firstly, applying the model to trip-level data and, secondly, by adding temperature and snow cover as explanatory factors. These two weather variables were found to best reflect the seasonal variations in cycling.

The cycling model was fitted to data from the Finnish National Travel Survey (NTS) for 2016, which was at the time the latest available [8]. The NTS is based on one-day travel diaries with over 30 000 survey respondents over the year. The same survey methods were used for both the national sample and ten regional samples aiming to create a closer look at urban travel. Our model for cycling behaviour was based on data from the ten city regions (Oulu, Turku, Päijät-Häme, Tampere, Helsinki, Eastern Uusimaa, Western Uusimaa, Riihimäki, Salo, and Joensuu) covered by the regional samples as well as the national sample. In the NTS, trips made by bicycle and e-bike are recorded together without specific information on the use of e-bikes. The data included 4,985 up to 30-kilometre-long trips from home to work by adults between the ages of 18 and 64. To simplify model building, these trips were further filtered to include only those with direct returns (work–home) or 3,677 trips. These trips formed the basis for our logistic regression model.

**Fig 2:**
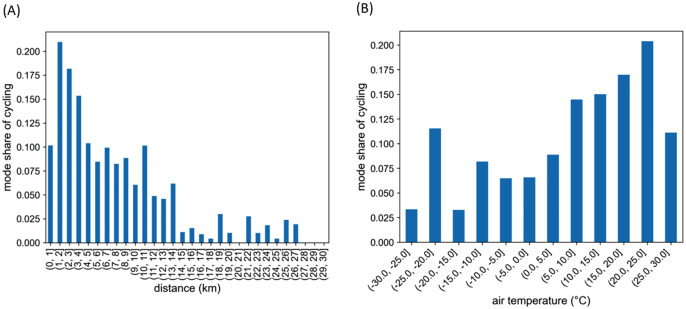
Mode share of cycling according to distance and temperature. Mode share of cycling on trips to and from work on distances ≤ 30 km according to (A) trip distance and (B) temperature.

**Fig 3:**
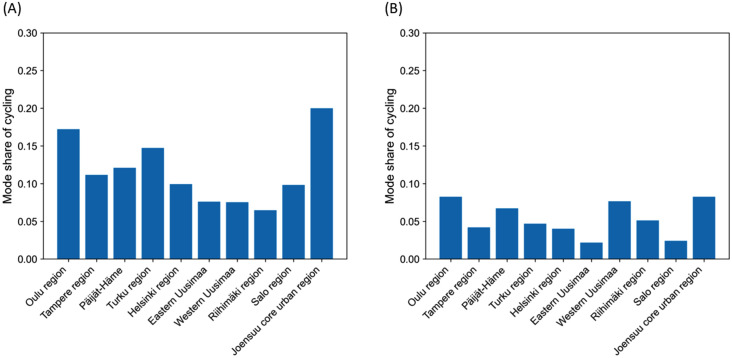
Seasonality of cycling in city regions. Mode share of cycling in Finnish city regions on trips to and from work for distances ≤ 30 km in **(A)** April–October and **(B)** November–March.

An analysis of the NTS data showed that the mode share of cycling on trips to work followed a long-tailed curve, with a peak around 1–3 kilometres (**Fig 2**). Differences between men and women were also observed, with women cycling more on short trips (up to two kilometres). The cycling of women also seemed more sensitive to winter conditions. This finding is corroborated by Kajosaari et al. [51]. It can be seen in the NTS data that cycling levels also vary significantly between different city regions (**Fig 3**). The NTS data also reveals that having a car at one’s disposal impacts cycling significantly.

To link the trips by bicycle to weather conditions, open weather data from the Finnish Meteorological Institute [52] was exploited. Data from the weather station closest to the respondent’s home at the start of the trip was joined with the NTS data. An initial data analysis showed that temperature and snow cover (a binary variable) had a major impact on cycling, whereas the impact of other weather variables (such as wind, visibility, and rain) was unclear. It has been shown in some studies that high temperatures (above +25°C) have a negative impact on cycling [38]. Although this was also observed in our data (**Fig 2**), there were very few observations for temperatures above +25°C.

**Fig 4:**
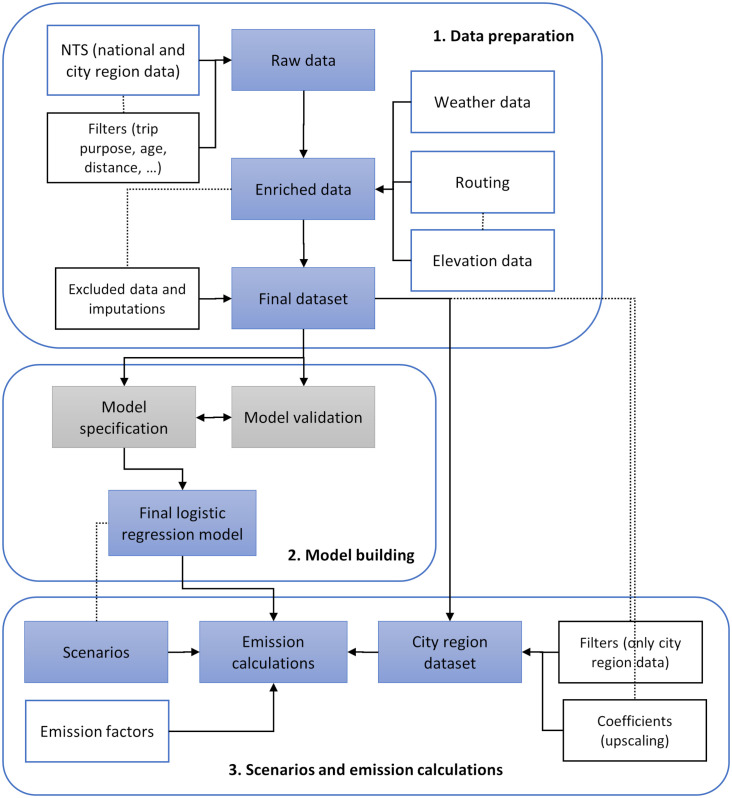
Modelling framework.

The geocoordinates for both the departure and arrival destinations in the NTS were available. This information was used to route the trips using the Digitransit router [53] and the OpenTripPlanner plugin of QGIS [54]. The Road Slope Calculator of QGIS [55] was exploited for calculating the average route gradient based on a Digital Elevation Model from the National Land Survey of Finland (contains data from the National Land Survey of Finland Topographic Database 01/2019) [56]. Missing gradient information was imputed with the median value (1.88%). After filtering out trips with missing weather information and trips where the routed distance was over 30 kilometres as well as one trip with a distance of zero, there were 3,409 commute trips left (Fig 4). Descriptive statistics for the key variables of the final dataset can be seen in S1 Appendix.

In this work, the aim was not to find all factors predicting cycling levels in Finland but to account for the major environmental factors that cannot be easily modified, such as distance, hilliness, and weather conditions. The aim was to arrive at a model that could be used to predict cycling in a realistic manner, given the current urban form and geography of Finnish cities. Variables for the different city regions were included as predictors as city regions display major differences in cycling (**Fig 3**). The city regions can also be taken as a proxy for cycling infrastructure, as this variable could not be included directly for lack of data. Gender and car availability (i.e., having a car always or nearly always at your disposal as a driver) were also included as explanatory variables so that the model fitted to the original observations from the NTS data would better reflect the behaviour of the population when upscaled to the level of the ten Finnish city regions. The coefficients provided in the NTS data were used to upscale the data to the city region level (see [16]). This upscaled data was used for estimating the overall mileage, replaced car travel, and climate emission reductions at city region level. The cycling uptake scenarios and emission calculations are described in the following sections. A schema for the modelling framework can be seen in **Fig 4**.

Predictors such as the total distance travelled during the day, maximal trip distance of the home-based trip chain, and accompanying other people were also considered as explanatory variables. They were however finally left out of the model as they did not increase its prediction accuracy.

### Cycling uptake scenarios

To explore the potential implications of changes in commuting behaviour, several cycling uptake scenarios were constructed. The ‘Oulu Fever’ scenario represents a case where people living in the ten city regions of the NTS data cycle like the residents of Oulu. In practice, this scenario describes a situation where cycling levels increase significantly and winter cycling gains in popularity. In addition, a speculative scenario, ‘Car-Free Living’, where everyone cycles similarly to commuters who do not have a car at their disposal, was included. Of course, not everyone owning and using a car is able to cycle, notably for various health-related reasons, care duties, or because they need a vehicle to transport work-related equipment. Nevertheless, we wished to explore the potential impacts of generalising the behaviour of the carless working population. Interestingly, results from the latest National Travel Survey from 2021 suggest that working-aged people who experience obstacles to mobility (notably difficulties to stand or walk, or poor physical condition) cycle as much as their counterparts [4].

We also wanted to explore the potential impact of e-bikes, as they have the potential to replace cars on longer trips and increase cycling frequency. There is also a company bike benefit scheme in Finland that is expected to further boost the demand for e-bikes. The construction of a scenario for e-bike adoption proved challenging as there is not, to our knowledge, any comprehensive data on the impact of electric bicycles on cycling behaviour in Finland. There have, however, been several studies on the impact of e-bikes on cycling behaviour in other countries. It has been shown in the Netherlands that e-bike owners cycle longer distances than regular cyclists [28]. Longer trip distances for e-bikes have also been observed in Switzerland [57] and in Norway [58]. It has been estimated in the Netherlands that the maximum acceptable distance for home-to-work commutes doubles in the case of e-bikes, reaching approximately 15 kilometres [59]. E-bikes might also mitigate the impact of route gradient: in a Norwegian survey, factors such as physical strain and hilliness were perceived as barriers to cycling by around 20% of the respondents [60] while in Sweden ‘less effort/increased comfort’ was cited as one of the main reasons for acquiring an e-bike [61]. The Swedish survey also suggests that e-bikes might make cycling on commute trips more resistant to winter conditions [61]. It has been confirmed by many surveys that while e-bikes substitute for conventional bicycles on some trips, they are also replacing other modes, notably car use (see, e.g., [28,58]). De Haas et al. [27] also found that e-biking replaced car use on commuting trips.

Information on how average trip distances increase due to e-bikes is not, however, very useful in our case, as the average distance depends largely on the length of trips in general. Information on how cycling probability or mode shares change in different distance bins is needed. This type of information is provided in Sun et al. [28], showing that with e-bikes the mode share of cycling is nearly the same for 5–10-kilometre trips as for trips of less than five kilometres. Based on this indication, the cycling probability in the e-bike scenarios was based on the baseline probability *pcycle* (see Equations 1 and 2 in Results), but the peak—attained at approximately 2.3 kilometres when there is no snow and at 2.2 kilometres in snowy conditions—was extended to twice that distance, thus becoming a plateau. The remainder of the baseline curve was shifted forward correspondingly. We call this new e-cycling probability *pcycle’*. Scenarios where e-bikes would remove the impact of the route gradient were also explored, one based on the modified probability *pcycle’* and another on the baseline probability *pcycle*. In the latter case it is therefore assumed that e-bikes would merely remove the impact of hilliness compared to the baseline situation. A combined e-bike and Oulu Fever scenario was also modelled. No specific scenarios were implemented with respect to the impact of e-bikes on cycling in winter for lack of data. The possible interaction between hilliness and distance was not explored for this same reason.

### Carbon emission calculations

The climate emission reductions for the scenarios were estimated based on the substitution of trips currently made by car with cycling (direct trips to and from work). The emission reductions were calculated using average emission factors (CO_2_eq/km) based on a forecast for the greenhouse gas emissions and total mileage of the Finnish car fleet over ten years from 2022 to 2031 [62]. The emission factor values can be found in S1 Appendix. This forecast represents the future greenhouse gas emissions of the Finnish car fleet based on the projected development of fleet size, its powertrains and electrification, energy efficiency, and changes in yearly mileage given currently implemented policy measures [62]. The emission factors only account for direct greenhouse gas emissions. Indirect and upstream emissions are not taken into account; for instance, emissions from electricity generation or the life-cycle emissions of biofuels are not included.

Emissions reductions were calculated based on replaced car mileage. Only travel by car as a driver was taken into account in our estimates. Car trips that were replaced by cycling were obtained by comparing the cycling probability in the scenarios with the baseline probability to cycle *pcycle* for trips currently made by car. The comparison of the scenarios to the baseline probability is necessary as the modelled probability to cycle predicted by the logistic regression model is never zero. The estimated emission reductions only consider car travel replaced by cycling and not the replacement of other modes (such as walking and public transport use). The electricity consumption of e-bikes and emissions from public transport were assumed to be insignificant compared to emissions from cars.

The emission reductions were estimated for urban commuting at the level of the ten Finnish city regions covered by the NTS data (Oulu, Turku, Päijät-Häme, Tampere, Helsinki, Eastern Uusimaa, Western Uusimaa, Riihimäki, Salo, and Joensuu). The emission reductions were estimated at the city region level instead of the national level as there is presumably less cycling infrastructure outside urban areas; this poses a practical obstacle to increasing cycling uptake. Lastly, a scaling factor of 1.11 (based on the upscaling coefficients of trips with missing weather and gradient information) was used to adjust the calculated emission reductions.

## Results

### Logistic regression model

**Fig 5:**
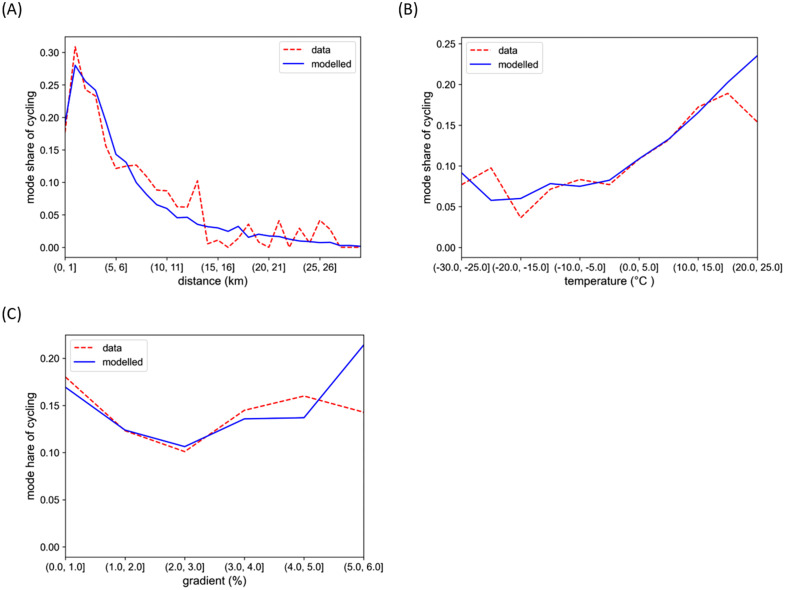
Comparison of model and data. Comparison of the proportion of cyclists in the NTS data and the modelled mode share for data binned by (A) distance, (B) temperature, and (C) gradient.

The final formulation of the logistic regression cycle model for commute trips in Finland is shown below (Equations 1 and 2). This model expresses the probability that a trip is cycled. **Table 1** shows the values of the β parameters (see S1 Appendix for a note on their interpretation). The explanatory factors linked to trip distance include non-linear terms, including a cubic one, in order to replicate the sharpness of the peak in cycling and its long-tailed curve observed in the NTS data (**Fig 5**). We did not include higher order polynomials to avoid overfitting. Binary variables for gender and the availability of a car were also included. The seasonality of cycling was taken into account through the inclusion of variables for temperature and snow. An interaction or a product term was also added to better describe the dependence between weather conditions (here the presence of snow) and trip length, as longer trips are cycled in summer conditions. In addition, an interaction term was added to reflect the fact that low temperatures affect the cycling of women differently than that of men. Other product terms were considered as well (notably between temperature and distance, gender and distance, gender and snow, men and car availability, and car availability for women) but they were finally excluded as their addition was not deemed to bring the model closer to the behaviour observed in the NTS data and to increase its classification accuracy.

**Table 1:** Parameters of the logistic regression model.

Variable	Parameter value	p-value	95% confidence intervals	
constant	−3.1675	0.0000*	−4.5037	−1.8312
distance	−1.8343	0.0000*	−2.6240	−1.0445
sqrt(distance)	4.7359	0.0000*	2.7940	6.6778
sq(distance)	0.0611	0.0015*	0.0235	0.0987
cb(distance)	−0.0010	0.0129*	−0.0018	−0.0002
gradient	−0.0857	0.2608	−0.2349	0.0636
temperature	0.0158	0.1417	−0.0053	0.0369
snow†	−0.2476	0.2497	−0.6691	0.1740
snow * distance	−0.0281	0.3238	−0.0839	0.0277
gender‡	−0.3457	0.0167*	−0.6287	−0.0626
gender * temperature	0.0379	0.0037*	0.0123	0.0635
car^x^	−1.2978	0.0000*	−1.5421	−1.0535
Oulu	0.8546	0.0000*	0.5157	1.1935
Helsinki	−0.4847	0.0071*	−0.8375	−0.1319
Joensuu	0.3778	0.2393	−0.2515	1.0072

*p < 0.05

^†^no snow equals 0, snow equals 1

^‡^man equals 0, woman equals 1

^x^no car at disposal equals 0, a car at disposal equals 1.

**Fig 6:**
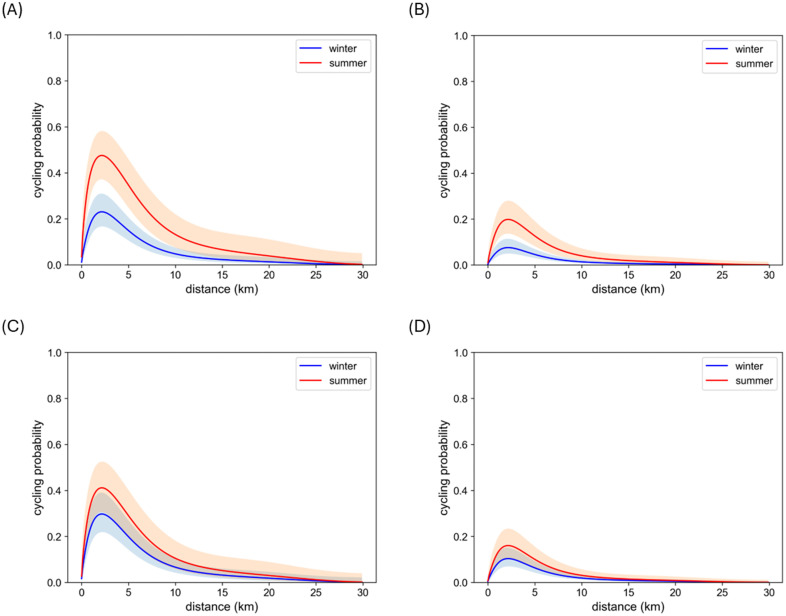
Modelled probability to cycle according to distance. Example of the modelled cycling probability and its 95% confidence intervals for the Helsinki region in summer and winter conditions corresponding to the average of min and max temperatures on Midsummer and Christmas Eves [64] or +16°C and 0°C without and with snow respectively and with a 2% gradient for (A) women with no car, (B) women with a car, (C) men with no car, and (D) men with a car.

The baseline probability to cycle or *pcycle* can now be expressed as follows:


$$\begin{array}{c} logit(cycle)=\;\beta_{\text{0}}+\beta_{\text{1}}*distance+\beta_{\text{2}}*\sqrt{distance}+\;\beta_{\text{3}}*{distance}^{\text{2}}\\ \;\;\;\;\;+\;\beta_{\text{4}}*{distance}^{\text{3}}+\beta_{\text{5}}*gradient+\beta_{\text{6}}*temperature+\beta_{\text{7}}*snow\\ \;\;\;\;\;+\;\beta_{\text{8}}*snow*distance+\beta_{\text{9}}*gender+\beta_{\text{1}\text{0}}*temperature*gender\\ \;\;\;\;\;+\;\beta_{\text{1}\text{1}}*car+\beta_{\text{1}\text{2}}\;*\;Oulu+\beta_{\text{1}\text{3}}\;*Helsinki+\;\beta_{\text{1}\text{4}}\;*\;Joensuu \end{array}$$ (1)

$$pcycle=\;\frac{\text{1}}{\text{1}+e^{-logit(cycle)}}$$ (2)

where *distance* is the trip distance (kilometres), *gradient* is the average trip gradient (%), *temperature* is the air temperature (°C), *snow* stands for the presence of snow (no snow equals 0, snow equals 1), *gender* denotes the cyclist’s gender (with the values 0 and 1 for individuals identified as men and women in the NTS data respectively), *car* stands for car availability or having a car always or nearly always at your disposal (0 for no and 1 for yes), and *Oulu*, *Helsinki*, and *Joensuu* are the dummy variables for the corresponding city region (with 1 for trips in the region in question and 0 otherwise). Other regional variables (for Turku, Päijät-Häme, Tampere, Eastern Uusimaa, Western Uusimaa, Riihimäki, and Salo) were excluded as they were not statistically significant. We chose to include the variable for Joensuu although its p-value was above 0.05 as it is a well-known cycling city in Finland and also stands out in the NTS data (**Fig 3**). In addition, the trip gradient was included as it has an impact on the physical effort required. Parkin et al. [63] found that hilliness was the most important physical variable impacting cycling to work. A comparison between the model and the NTS data is seen in **Fig 5**. The AUC calculated for the model is approximately 0.82 and the accuracy score 0.88, with more false negatives than positives, when trips with a predicted probability to cycle greater than 0.50 are classified as cycled. An example of the modelled cycling probability for the Helsinki region can be seen in Fig 6. See S1 Appendix for more information on the marginal effects of the independent variables.

Our cycling model reveals two interesting interactions linked to cold climate winter conditions that have so far been little discussed in literature: 1) cycled distances tend to be shorter in winter conditions and 2) women’s cycling levels drop more drastically in winter than those of men. The first observation could be linked both to the increased effort required when cycling in snow and to increased risk levels (see, e.g., [65]) due to icy and slippery roads. With regards to gender differences, this phenomenon could be linked to the fact that women report typically report higher levels of perceived risk than men (see, e.g., the discussion in [66]). These observed interactions are consistent with the sparse literature available. In Sweden, a seasonal association between commute cycling and trip distances has been observed, with nearly all bicycle trips longer than ten kilometres shifting to other modes in winter [46]. In Toronto, it has been observed that women’s propensity to cycle is more negatively affected by low temperatures than that of men [67] and that women are less likely to be all-year cyclists, though no association between commute distances and seasonal variation in cycling was found [68]. It has also been observed in Norway that women cycle less in winter than men when controlling for cycling generally [69], and in Finland both older and female cyclists were found to be more likely to decrease cycling in winter than their younger or male counterparts [51]. Both of these phenomena—associations between gender and winter cycling, and winter cycling and shorter distances cycled—are relevant for an accurate assessment of year-round cycling potential in countries with cold and snowy winters.

### Scenarios and emission calculations

The scenarios are summarized in **Table 2**. The predicted cycling probability has been interpreted here as a fraction of trips by bicycle. In the scenarios, the new cycling probability is calculated for all trips. In the Oulu Fever scenario, the regional dummy for Oulu is set to 1 for all commute trips and the values of all the other regional variables are set to 0. In the Car-Free Living scenario, the *car* variable is set to 0 (no car available) and in the e-bike scenarios 2 and 3 *gradient* is set to 0 for all trips (see Equation 1).

**Table 2:** Cycling uptake scenarios.

Scenario	Description	Probability to cycle
Baseline	Baseline scenario	*pcycle* (see Equations 1 and 2)
Oulu Fever	Everyone cycles like people in the Oulu region	*pcycle* with *Oulu* = 1 and other regional dummy variables = 0
Car-Free Living	Everyone cycles like people without a car	*pcycle* with *car* = 0
E-bikes variant 1 (v1)	E-bikes extend the distance range of cycling	*pcycle’* (see Cycling uptake scenarios)
E-bikes variant 2 (v2)	E-bikes extend the distance range of cycling and remove the impact of hilliness	*pcycle’* with *gradient* = 0
E-bikes variant 3 (v3)	E-bikes remove the impact of hilliness	*pcycle* with *gradient* = 0
E-bikes v1 & Oulu Fever	Everyone cycling like people in the Oulu region combined with e-bikes extending the distance range of cycling	*pcycle’* with *Oulu *= 1 and other regional dummy variables = 0

**Fig 7:**
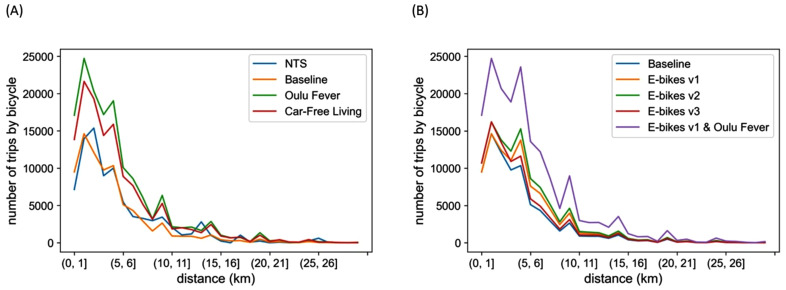
Number of commute trips cycled per day for the cycling uptake scenarios. The number of trips is calculated by distance bin.

The results for the cycling uptake scenarios in terms of number of trips by bicycle, cycled mileage, substitution of travel by car, and resulting emission reductions for car use between home and work on trips under 30 kilometres are given in Table 3 (see S1 Appendix for detailed results). In our estimates trips by bicycle include also those made with an e-bike. The number of trips cycled by distance bin is presented in **Fig 7**. In Table 3 and in Fig 7, the values for National Travel Survey correspond to the observations in the NTS dataset whereas the other rows/lines represent simulation results for the scenarios. The cycling uptake scenarios are compared against the baseline scenario.

**Table 3:** Emission reductions and other results by cycling uptake scenario.

Scenario	Number of trips cycled (trips/day)*	Mileage cycled (Mkm/day)	Replaced car trips as driver (trips/day)	Emission reduction (%)†	Emission reduction over 5–10 years (ktCO_2_eq)
National Travel Survey	86,000	0.46	NA	NA	NA
Baseline	79,000	0.37	NA	NA	NA
Oulu Fever	150,000	0.77	21,000	3.7%	34–58
Car-Free Living	130,000	0.68	33,000	6.0%	55–94
E-bikes v1	95,000	0.49	6,500	1.3%	12–21
E-bikes v2	110,000	0.56	10,000	2.0%	18–32
E-bikes v3	89,000	0.42	3,100	0.5%	5–8
E-bikes v1 & Oulu Fever	170,000	0.98	32,000	6.1%	56–96

*Both trips to and from work are included

^†^Emission reductions are calculated with respect to emissions from car use as driver on direct trips between home and work of maximum 30 kilometres

In the Oulu Fever scenario, the number of trips by bicycle nearly doubles and the cycled mileage more than doubles compared to the baseline scenario (+88% and +110% respectively). The Oulu Fever scenario brings an emission reduction of 3.7%, or approximately 34 ktCO_2_eq over five years. The Car-Free Living scenario is interesting as well: although the number of trips cycled and their mileage is smaller than in the Oulu Fever scenario, the obtained emission reductions are higher (−6.0%) as in this scenario as more car use gets replaced with cycling. The first variant of the e-bike scenarios brings a modest emission reduction (−1.3%). The second variant of the e-bike scenario where hilliness no longer hinders cycling brings a somewhat larger emission reduction (−2.0%). The third e-bike scenario is the most conservative one and only brings an emission reduction of 0.5%. This means that hilliness alone has little impact on cycling at the city region level. The combination of the Oulu Fever scenario with e-bikes suggests that cycling levels could more than double (+120%) in a favourable context and that cycled mileage might increase by over 160% compared to the baseline scenario. This scenario also brings the greatest emission reduction or 56 ktCO_2_eq over five years (−6.1%).

When looking at cycling potential in terms of number of trips in different travel-related urban zones [70], cycling potential is found in nearly all types of urban zones, from walkable city centres and their fringes to surrounding public transport and car-based zones. The greatest emission reduction potential is however found in the public transport and car zones, where the most potential for increasing cycling mileage is found and where the mode share of driving is also high. For more information on the concept of urban fabrics and travel-related urban zones see Helminen et al. [70].

### Illustration of cycling potential on a map

**Fig 8:**
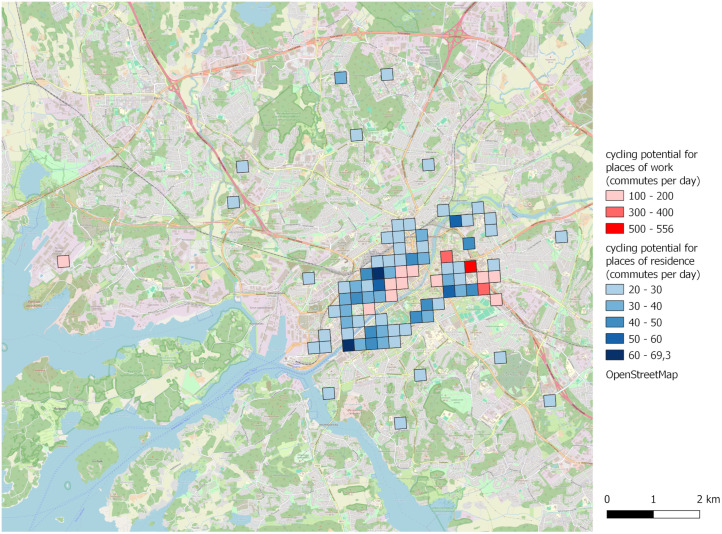
Commute cycling potential in Turku. Example of commute cycling potential based on the workplace (in red) and residential (in blue) locations on a 250 m x 250 m grid. Locations with a cycling potential greater than 100 and 20 daily commutes are shown for workplaces and places of residence respectively. Assumptions: summer conditions (+15°C, no snow), 2% gradient, and car available. The calculations are based on employment statistics for 2022 (YKR/ Finnish Environment Institute and Statistics Finland 2024) [71]. Contains information from OpenStreetMap and OpenStreetMap Foundation, which is made available under the Open Database License.

The cycling model developed here is also a useful tool for examining the spatial distribution of commute cycling potential in Finland’s city regions. For instance, the application of the model to the city region of Turku using employment statistics (origin–destination data) from Statistics Finland on a 250 m x 250 m grid [71] allows to highlight the locations where the greatest potential for cycling is likely to be found based on commute distances, the predicted probability to cycle, and the number of employed people potentially travelling to work (**Fig 8**). The potential can be explored either based on workplaces or residential areas to find suitable targets for intervention. Of course, remote working, existing cycling infrastructure, and the current modal share of active travel, among others, would also need to be considered when planning interventions. In Turku, while most of the workplaces where cycling potential is concentrated are found along the river in the centre of the city, there are also a few potential locations outside it, notably in the shipyard area (westwards) and towards Kupittaa (slightly east of the centre). In terms of residential locations, commute cycling potential is less concentrated, though a large part of it is located around the centre. This might justify rethinking the allocation of urban space to better accommodate cycling in the city centre.

## Discussion

### Policy implications

Based on our calculations, it would be possible to set relatively ambitious targets for increasing cycling in Finland. The Oulu Fever scenario illustrates that it would be possible to cycle significantly more on trips to work in Finland (88% more trips and 110% more mileage compared to the baseline scenario), even when considering real-life trip distances, hilliness, and weather conditions. A potential national target could be to replicate the cycling habits seen in the top cycling cities, Oulu and Joensuu, all over the country. Replicating the favourable conditions in Oulu would however require investments in cycling infrastructure and winter maintenance. Poor road maintenance was identified as one of the major obstacles to winter cycling by Kajosaari et al. [51]. The density of cycling infrastructure was also found to be positively associated with all-season cycling in Toronto [68]. On a general level, our results (and the example of Oulu) indicate that a cold climate is not an absolute obstacle to cycling and that the potential to increasing cycling uptake is considerable even in cold climate countries.

When the emission reductions are summed over several years, the order of magnitude of the results obtained in the Car-Free Living and the combined e-bike and Oulu Fever scenarios over ten years is similar to the estimated impacts of several of the major actions (e.g., development of public alternative fuels infrastructure, the Clean Vehicles Directive, and a purchase subsidy for electric trucks) listed in the Finnish Roadmap to fossil-free transport [2]. In the Roadmap, investments in active travel infrastructure (both walking and cycling) were estimated to bring emission savings of 4–15 ktCO_2_ till 2030 [2]. The upper limit of this estimate, or 15 kilotonnes, is less than a half of the emission reductions from the Oulu Fever scenario over five years. Given that these estimates from the Roadmap date back several years, the emission factors used were probably higher than ours, leading also to higher emission reductions. It would therefore seem that cycling has been underestimated as a means to bring emission benefits and to contribute to achieving the national carbon neutrality target.

The e-bike scenarios explored in this work are speculative as we lack trip-level data for building a scenario in a similar manner to the Oulu Fever one. The obtained results are however promising in terms of the emissions reduction potential of electric bicycles. Survey findings from the Netherlands, Norway, and Sweden have shown the mode share of cycling more than doubling thanks to e-bikes [28,58,59]. The conclusions of the e-bike scenarios are strengthened by the Car-Free Living scenario which depicts, based on working adults that do not generally have a car at their disposal, people cycling more and longer trip distances. Based on these results, it would be worthwhile to consider what kind of results might be obtained if e-bikes were promoted more systematically. In Norway, a subvention scheme for e-bikes resulted in an increase in the mode share of cycling and more cycling activity [72]. Rural areas and the rural–urban fringe also constitute an excellent target where the promotion of e-bikes might bring significant benefits in terms of transport decarbonisation [21]. The potential to substitute trips made by car with e-biking in this context would need to be further explored in Finland where population densities and the size of rural areas differ from those in central Europe.

Though e-bikes might bring more sizeable emission benefits than estimated here, the estimated emission savings remain relatively modest (at most only about 1.2%) when considering all car use on trips to and from work and not just distances up to 30 kilometres. This is in large part due to the limited distance range of (e-)cycling. Improving the combined use of bicycles and trains (or bike–train commuting) offers a further possibility to extend the range of cycling and to provide an attractive alternative to car use on interurban trips [73]. In Finland, less than 5% of cycled mileage currently comes from intermodal journeys [12]. Our model for cycling to work confirms unsurprisingly that trip distance has a strong impact on the cycling probability. This means that greater density and mixed land use, when leading to shorter trip distances, would likely have a positive impact on cycling potential. Commute distances have however been slowly increasing in Finland since 2010, stabilising at 15 kilometres [11]. In Finnish city regions, the potential for the use of sustainable commute modes has decreased in the last 35 years, stabilising by 2015, estimated based on the development of urban structure and home–work distances [11].

Given the health benefits of cycling as well as its other positive impacts on liveability in cities (less air pollution, noise, and congestion, more space for other travel modes, etc.), it would be interesting to promote cycling even when not considering climate emission benefits. Besides those who do not have a car at their disposal, there is another population group that displays even higher levels on cycling in Finland, at least on short trips: school children [45]. As a thought experiment, if we assume that adults would cycle like 12-year-old children on trips up to five kilometres (according to the model in [45]), the number of trips by bicycle would increase by 53% and the cycled mileage by over 26%. It has been estimated in Finland that the cost of low physical activity is €3.2 billion per year [74]. This would likely make investments in cycling infrastructure, improved winter maintenance, and subventions for e-bikes highly cost-effective from a public health perspective. It has been shown elsewhere that the benefits of investing in active travel are greater than its costs (see, e.g., [75–77]). It has been estimated at the EU-level that active travel could bring important savings given the external costs of motorised transport [78].

There are many policy measures that have been shown to increase cycling. Besides better cycling infrastructure and other ‘carrots’, Xiao et al. [79] have shown that the use of ‘sticks’ (such as reduced road space for cars, reduced or more expensive parking, road use charges, and traffic restrictions) and combined interventions can be more effective. Kiviluoto et al. [80] underline the importance of an integrated perspective, with changes in urban transport planning, infrastructure funding, and less car-centricity helping to promote active travel and more physically active lifestyles. It has also been underlined in literature that cycling is an integral part of sustainable transport systems and new urban models [81]. The example of European cities such as Paris demonstrates that change is possible, and an increasing number of cities are becoming cycling cities. In city centres, notably in Helsinki, increasing the mode share of cycling would likely require a reallocation of existing road space that has so far proven politically difficult in Finland. As national funding for the development of cycling infrastructure is being scaled down, it therefore seems doubtful that we will see major increases in cycling uptake in the near future.

### Limitations

Despite our best efforts to realistically represent cycling on trips to work, there are several caveats linked to the cycling model and emission calculations presented here. For instance, there are observable differences in the cycling habits of men and women, even when accounting for different trip distances, with women having a higher probability to cycle very short trips. This does not entirely come across in our model. Overall, the uncertainties linked to the modelled cycling probabilities are relatively large. In addition, aspects such as safety, perceived safety, and cycling infrastructure were not included as explanatory factors in our model for lack of data. As our aim was explorative and comparative, the model was however deemed functional for its purpose. While the results are generalisable to other countries and regions with cold weather, differences in baseline cycling levels, infrastructure, safety, and other key aspects may limit comparability.

The results obtained at the city region level are generally rather conservative as our model and scenarios tend to underestimate the probability to cycle and cycled mileage. When looking at trips in different distance bins, the baseline probability to cycle predicted by our model overestimates cycling on trips up to two kilometres and underestimates it for trips between two to three kilometres; thus, these differences somewhat cancel out overall. As our model only covers ten major city regions, there would likely be more emission gains to be made in various other urban regions and rural areas in Finland. Around 54% of the Finnish population lives in these ten city regions [16]. Given the insufficient coverage of the NTS data, we did not attempt to quantify cycling potential elsewhere. It should also be noted that in the NTS data the number of observations in some of the city regions, notably Joensuu, is limited (see S1 Appendix). In addition, this work only focuses on cycling to work. Commute trips represent around 15% of all domestic trips [15].

There are important uncertainties linked to the future emission factors of Finland’s car fleet used in estimating the emission impacts of increased cycling. The projections for the emissions of the Finnish vehicle fleet have been evolving rapidly in recent years, notably due to the speed of electrification but also due to changes in the national distribution obligation of biofuels. This limits the comparability of our calculations with past estimates for other decarbonisation measures, as the emission factors used are likely different—this can also lead to major differences in the estimated emission savings. While the focus has of this work been on climate impacts, increased cycling would have multiple co-benefits from positive impacts on air quality and noise pollution to decreasing resource use and increasing liveability in urban areas. This means that increasing cycling uptake remains relevant even as more and more cars become electric.

The investments in cycling infrastructure required for spreading the cycling levels of Oulu around Finland were not quantified in this work. It would be interesting to estimate the costs of the required infrastructure and additional winter maintenance to better quantify the cost-effectiveness of cycling in reducing transport emissions. It would also be useful to better understand how Oulu has conserved its high cycling levels and whether there are any local traits that might be difficult to replicate elsewhere. One study showed that despite increasing density and diversity in the inner urban area, even Oulu struggles with limiting car traffic [82].

The e-bike scenarios are speculative, as there is yet no trip-level data on the behaviour of e-cyclists in Finland. In the future it would be highly useful to collect data on the use of e-bikes in Finland to see how they modify the length of trips and the overall mileage, whether they have a positive impact on winter cycling, and whether they can entice more people to cycle. There might also be an interaction between gradient and trip distance; this was not taken into account in our e-bike scenarios. Trip chaining and its impacts on cycling is another topic that would merit further study. Combining cycling with public transport, notably trains, might also have considerable potential for replacing car travel and could be explored in a future study. In addition, public transport can be complementary to cycling on days when the weather is poor—the potential of this type of multimodal mobility could be explored in future work.

A new full-year National Travel Survey is now being prepared and when its results become available it will be interesting to see if any changes in cycling behaviour have taken place. The results of the latest part-year survey from 2024 [83] suggests that Finland has not experienced the kind of cycling renaissance seen in many other places. Though the impact of factors such as trip distance and gradient is also linked to the quality of cycling infrastructure, it is unlikely that major shifts in cycling behaviour (as depicted in our model) have taken place. A recent study for Helsinki showed a worrying overall decline in cycling traffic despite an increase on one new cycling highway [84]. While there have been long-term changes in commuting behaviour—remote working remains more prevalent than before Covid-19—it is unclear how this might impact our estimates, as working from home is presumably more prevalent amongst those with long commutes.

## Conclusions

This work modelled cycling on trips to work in Finland using binary logistic regression and explored scenarios for increased cycling uptake along with their climate emission impacts. The emission impacts were estimated at the level of the ten Finnish city regions covered by the National Travel Survey 2016. The modelled cycling behaviour accounted for trip distance and hilliness, as well as weather conditions (temperature and snow) that are a major determinant of cycling in this northern country. Our results indicate that in a scenario where all commuters cycle like the residents of the Oulu region, the top cycling city in Finland, we would see approximately 88% more trips by bicycle and a 110% increase in cycled mileage compared to the baseline. This Oulu Fever scenario would bring a 3.7% reduction in emissions from car use on direct commute trips up to 30 kilometres (or 34 ktCO_2_eq over five years). E-bikes also offer an impactful way to replace commute trips made by car as they extend the range of cycling. For a combined e-bike and Oulu Fever scenario, the resulting emission reductions (56 ktCO_2_eq when summed over a period of five years) become non-negligible when compared to other transport decarbonisation measures. Our results therefore indicate that it would be feasible to set an ambitious national target for cycling, even aiming to double the number of trips by bicycle or the cycled mileage.

## Supporting information

S1 File Appendix. (DOCX)
