# Supplementary material for: The potential of bicycle commuting to reduce carbon emissions in Finland
Source: PLoS One. 2025 Nov 13;20(11):e0335010. doi: 10.1371/journal.pone.0335010 (PMC12614520; doi:10.1371/journal.pone.0335010)
Supplement: S1 File — (DOCX) [file pone.0335010.s001.docx]

S1 Appendix

**Descriptive statistics**

*Table S1.1 Descriptive statistics for continuous variables in the final dataset*

| **Variable** | **Unit** | **Mean** | **Standard deviation** | **Min** | **Median** | **Max** |
| --- | --- | --- | --- | --- | --- | --- |
| distance | km | 10.03 | 8.06 | 0.02 | 8.0 | 30.0 |
| gradient | % | 1.88 | 0.76 | 0.18 | 1.87 | 6.86 |
| temperature | °C | 3.91 | 9.34 | -33.8 | 3.1 | 26.7 |
| age | years | 43.01 | 12.16 | 18 | 44 | 64 |

*Table S1.2 Frequencies of categorical variables in the final dataset*

| **Variable** | **Value** | **Frequency** | **Percentage** |
| --- | --- | --- | --- |
| trip cycled | yes | 424 | 12.4% |
|  | no | 2985 | 87.6% |
| gender | man | 1736 | 50.1% |
|  | woman | 1673 | 49.1% |
| snow | yes | 1705 | 50.0% |
|  | no | 1704 | 50.0% |
| car available | yes | 2667 | 78.2% |
|  | no | 742 | 21.8% |
| city region | no city region | 619 | 18.1% |
|  | Oulu | 430 | 12.6% |
|  | Tampere | 451 | 13.2% |
|  | Päijät-Häme | 302 | 8.9% |
|  | Turku | 485 | 14.2% |
|  | Helsinki | 565 | 16.6% |
|  | Eastern Uusimaa | 145 | 4.3% |
|  | Western Uusimaa | 147 | 4.3% |
|  | Riihimäki | 55 | 1.6% |
|  | Salo | 121 | 3.5% |
|  | Joensuu | 90 | 2.6% |

**Emission factors**

*Table S1.3 Emission factors for car kilometres [1]*

| **Year** | **Emission factor (gCO_2_eq/km)** |
| --- | --- |
| 2022 | 132 |
| 2023 | 126 |
| 2024 | 110 |
| 2025 | 105 |
| 2026 | 100 |
| 2027 | 94 |
| 2028 | 88 |
| 2029 | 82 |
| 2030 | 76 |
| 2031 | 70 |
| 2032 | 65 |
| 2033 | 59 |
| 2034 | 54 |
| 2035 | 49 |

**Marginal effects**

For product terms, the sign of the coefficient in a logistic regression model does not necessarily describe the interaction accurately [2], [3]. In addition, in logistic regression the statistical significance of the interaction term is neither a necessary nor a sufficient condition for a ‘substantively meaningful’ interaction between the independent variables [2]. The magnitudes of the independent variables in logistic regression models can be explored by calculating their marginal effects (Table S2.4). Marginal effects can be used to compare how the predicted probability is impacted by a change in value of the explanatory variables [3].

An analysis of the marginal effects at the mean shows that the availability of a car has a much stronger negative impact than the presence of snow or gender (−0.111660, −0.019952 and −0.008598 respectively). For the city regions, Oulu, Helsinki, and Joensuu have the marginal effects of 0.060141, −0.020042 and 0.021339 respectively. For the continuous variables, the impact of a one-kilometre change in trip distance (−0.008230) is stronger than the change of one degree for gradient (−0.004072). A significant change in temperature can also have a similar impact (0.00753 for a 10-degree change in temperature).

*Table S1.4 Marginal effects (* p < 0.05)*

| **Variable** | **Contrast** | **Marginal effect at the mean (MEM)** | **p-value (MEM)** | **Average marginal effect (AME)** | **p-value (AME)** |
| --- | --- | --- | --- | --- | --- |
| distance | dy/dx | −0.008230 | < 0.001* | 0.00556 | 0.00586* |
| gradient | dy/dx | −0.004072 | 0.27205 | −0.00766 | 0.26050 |
| temperature | dy/dx | 0.000753 | 0.12077 | 0.00318 | < 0.001* |
| snow | 1–0 | −0.019952 | 0.02000* | −0.03414 | 0.01903* |
| gender | 1–0 | −0.008598 | 0.10798 | −0.00962 | 0.35976 |
| car | 1–0 | −0.111660 | < 0.001* | −0.13978 | < 0.001* |
| Oulu | 1–0 | 0.060141 | < 0.001* | 0.09024 | < 0.001* |
| Helsinki | 1–0 | −0.020042 | 0.00411* | −0.03952 | 0.00286* |
| Joensuu | 1–0 | 0.021339 | 0.31166 | 0.03699 | 0.27882 |

**Results by scenario**

*Table S1.5 Trips cycled (trips per day) by distance bin on trips between home and work in the ten city regions for the baseline (BSL), Oulu Fever (OF), Car-Free Living (CFL), e-bikes variant x (EBx), and e-bikes and Oulu Fever (EBO) scenarios*

|  | **All trips  (per day)** | **Trips cycled (per day)** | | | | | | | |
| --- | --- | --- | --- | --- | --- | --- | --- | --- | --- |
| **Distance bin (km)** | **NTS** | **NTS** | **BSL** | **OF** | **CFL** | **EB1** | **EB2** | **EB3** | **EBO** |
| (0, 1] | 46 603 | 6 907 | 9 490 | 17 096 | 13 846 | 9 490 | 10 700 | 10 700 | 17 096 |
| (1, 2] | 52 493 | 14 081 | 14 621 | 24 733 | 21 645 | 14 621 | 16 214 | 16 214 | 24 733 |
| (2, 3] | 44 751 | 15 891 | 12 099 | 20 309 | 19 319 | 12 396 | 13 786 | 13 469 | 20 692 |
| (3, 4] | 38 850 | 9 094 | 9 763 | 17 192 | 14 385 | 11 057 | 12 288 | 10 909 | 18 882 |
| (4, 5] | 49 908 | 10 006 | 10 343 | 19 034 | 15 890 | 13 759 | 15 279 | 11 616 | 23 586 |
| (5, 6] | 32 190 | 5 410 | 5 115 | 10 083 | 8 897 | 7 600 | 8 606 | 5 886 | 13 582 |
| (6, 7] | 34 996 | 3 520 | 4 325 | 8 606 | 7 662 | 6 602 | 7 416 | 4 910 | 12 205 |
| (7, 8] | 26 366 | 3 268 | 2 932 | 6 115 | 5 197 | 4 548 | 5 150 | 3 359 | 8 674 |
| (8, 9] | 19 632 | 2 852 | 1 578 | 3 162 | 3 154 | 2 439 | 2 812 | 1 839 | 4 599 |
| (9, 10] | 41 454 | 3 440 | 2 648 | 6 350 | 5 274 | 3 972 | 4 622 | 3 107 | 8 974 |
| (10, 11] | 16 660 | 2 062 | 907 | 2 143 | 1 857 | 1 307 | 1 512 | 1 054 | 2 983 |
| (11, 12] | 19 938 | 1 048 | 875 | 2 012 | 2 014 | 1 218 | 1 417 | 1 021 | 2 708 |
| (12, 13] | 19 149 | 1 182 | 871 | 2 102 | 1 767 | 1 162 | 1 347 | 1 013 | 2 720 |
| (13, 14] | 18 131 | 2 773 | 601 | 1 639 | 1 356 | 785 | 916 | 703 | 2 083 |
| (14, 15] | 38 043 | 1 522 | 1 048 | 2 853 | 2 449 | 1 321 | 1 560 | 1 240 | 3 530 |
| (15, 16] | 14 372 | 230 | 401 | 1 015 | 900 | 493 | 579 | 473 | 1 222 |
| (16, 17] | 10 712 | 0 | 257 | 675 | 651 | 311 | 366 | 304 | 804 |
| (17, 18] | 13 042 | 1 026 | 289 | 712 | 762 | 346 | 402 | 335 | 846 |
| (18, 19] | 4 330 | 78 | 55 | 164 | 176 | 66 | 80 | 66 | 198 |
| (19, 20] | 23 606 | 234 | 484 | 1 348 | 1 008 | 593 | 701 | 573 | 1 628 |
| (20, 21] | 5 693 | 0 | 85 | 260 | 185 | 106 | 128 | 102 | 322 |
| (21, 22] | 10 885 | 80 | 150 | 370 | 411 | 196 | 227 | 174 | 480 |
| (22, 23] | 3 874 | 0 | 29 | 66 | 98 | 40 | 46 | 33 | 91 |
| (23, 24] | 3 365 | 48 | 24 | 52 | 80 | 35 | 41 | 28 | 77 |
| (24, 25] | 19 726 | 238 | 160 | 407 | 425 | 252 | 295 | 188 | 630 |
| (25, 26] | 4 570 | 611 | 44 | 144 | 69 | 73 | 85 | 51 | 232 |
| (26, 27] | 4 587 | 42 | 38 | 106 | 84 | 69 | 81 | 45 | 185 |
| (27, 28] | 6 086 | 0 | 12 | 34 | 38 | 26 | 30 | 15 | 71 |
| (28, 29] | 1 183 | 0 | 3 | 8 | 11 | 7 | 8 | 3 | 18 |
| (29, 30] | 10 283 | 0 | 19 | 57 | 36 | 52 | 62 | 23 | 156 |

*Table S1.6 Distance cycled (km per day) by distance bin on trips between home and work in the ten city regions for the baseline (BSL), Oulu Fever (OF), Car-Free Living (CFL), e-bikes variant x (EBx), and e-bikes and Oulu Fever (EBO) scenarios*

|  | **Total  distance  (km)** | **Distance cycled (km)** | | | | | | | |
| --- | --- | --- | --- | --- | --- | --- | --- | --- | --- |
| **Distance bin (km)** | **NTS** | **NTS** | **BSL** | **OF** | **CFL** | **EB1** | **EB2** | **EB3** | **EBO** |
| (0, 1] | 32 438 | 5 687 | 7 274 | 12 963 | 10 659 | 7 274 | 8 201 | 8 201 | 12 963 |
| (1, 2] | 91 979 | 24 022 | 26 025 | 43 760 | 38 343 | 26 025 | 28 867 | 28 867 | 43 760 |
| (2, 3] | 124 367 | 43 607 | 33 294 | 55 921 | 53 740 | 34 171 | 38 010 | 37 072 | 57 054 |
| (3, 4] | 146 510 | 32 537 | 36 302 | 64 239 | 53 798 | 41 256 | 45 847 | 40 558 | 70 751 |
| (4, 5] | 242 394 | 47 461 | 49 861 | 92 022 | 76 947 | 66 485 | 73 838 | 56 006 | 114 201 |
| (5, 6] | 187 741 | 31 735 | 29 821 | 58 721 | 51 845 | 44 334 | 50 191 | 34 305 | 79 145 |
| (6, 7] | 240 501 | 23 295 | 29 577 | 58 928 | 52 499 | 45 192 | 50 769 | 33 589 | 83 645 |
| (7, 8] | 207 485 | 25 719 | 23 029 | 47 999 | 40 889 | 35 719 | 40 451 | 26 394 | 68 094 |
| (8, 9] | 173 078 | 24 442 | 13 922 | 27 793 | 27 866 | 21 507 | 24 796 | 16 224 | 40 424 |
| (9, 10] | 411 939 | 33 757 | 26 285 | 63 091 | 52 304 | 39 430 | 45 892 | 30 849 | 89 155 |
| (10, 11] | 181 441 | 22 583 | 9 880 | 23 323 | 20 205 | 14 233 | 16 465 | 11 481 | 32 465 |
| (11, 12] | 238 831 | 12 578 | 10 487 | 24 125 | 24 131 | 14 606 | 16 988 | 12 245 | 32 465 |
| (12, 13] | 246 611 | 15 336 | 11 254 | 27 153 | 22 789 | 15 013 | 17 399 | 13 089 | 35 131 |
| (13, 14] | 250 874 | 37 593 | 8 281 | 22 565 | 18 765 | 10 814 | 12 610 | 9 677 | 28 672 |
| (14, 15] | 568 900 | 22 586 | 15 686 | 42 722 | 36 654 | 19 776 | 23 347 | 18 557 | 52 844 |
| (15, 16] | 228 955 | 3 686 | 6 379 | 16 114 | 14 353 | 7 831 | 9 200 | 7 511 | 19 414 |
| (16, 17] | 181 931 | 0 | 4 365 | 11 462 | 11 048 | 5 274 | 6 219 | 5 156 | 13 661 |
| (17, 18] | 233 999 | 18 461 | 5 167 | 12 742 | 13 676 | 6 200 | 7 196 | 6 003 | 15 149 |
| (18, 19] | 81 059 | 1 452 | 1 024 | 3 066 | 3 307 | 1 246 | 1 498 | 1 232 | 3 708 |
| (19, 20] | 471 890 | 4 683 | 9 682 | 26 963 | 20 148 | 11 863 | 14 018 | 11 459 | 32 555 |
| (20, 21] | 118 895 | 0 | 1 772 | 5 432 | 3 872 | 2 223 | 2 671 | 2 131 | 6 739 |
| (21, 22] | 238 811 | 1 702 | 3 291 | 8 117 | 9 025 | 4 304 | 4 986 | 3 815 | 10 532 |
| (22, 23] | 88 879 | 0 | 664 | 1 513 | 2 248 | 922 | 1 062 | 766 | 2 099 |
| (23, 24] | 80 691 | 1 122 | 567 | 1 255 | 1 915 | 833 | 972 | 662 | 1 836 |
| (24, 25] | 493 027 | 5 958 | 4 008 | 10 169 | 10 617 | 6 296 | 7 374 | 4 700 | 15 757 |
| (25, 26] | 117 757 | 15 462 | 1 122 | 3 706 | 1 782 | 1 863 | 2 185 | 1 318 | 5 957 |
| (26, 27] | 122 881 | 1 147 | 1 019 | 2 814 | 2 242 | 1 833 | 2 139 | 1 193 | 4 893 |
| (27, 28] | 169 729 | 0 | 339 | 943 | 1 044 | 712 | 847 | 404 | 1 966 |
| (28, 29] | 34 318 | 0 | 88 | 223 | 320 | 212 | 239 | 100 | 532 |
| (29, 30] | 308 441 | 0 | 570 | 1 722 | 1 091 | 1 570 | 1 875 | 681 | 4 683 |

**References**

[1] Markkanen K, Lauhkonen A, Niemi A. Scenarios for greenhouse gas emissions and energy consumption of road transport in Finland: Exploring the impact of existing policies. VTT Technical Research Centre of Finland; 2023. VTT Technology 413. <https://doi.org/10.32040/2242-122X.2023.T413>

[2] Berry WD, DeMeritt JHR, Esarey J. Testing for Interaction in Binary Logit and Probit Models: Is a Product Term Essential? Am J of Political Sci. 2010;54:248–266. <https://doi.org/10.1111/j.1540-5907.2009.00429.x>

[3] Mize T. Best Practices for Estimating, Interpreting, and Presenting Nonlinear Interaction Effects. Sociol Sci. 2019;6(February):81–117. <https://doi.org/10.15195/v6.a4>
